# Risk Factors For Stroke, Myocardial Infarction, or Death Following Carotid Endarterectomy: Results From the International Carotid Stenting Study

**DOI:** 10.1016/j.ejvs.2015.08.006

**Published:** 2015-12

**Authors:** D. Doig, E.L. Turner, J. Dobson, R.L. Featherstone, G.J. de Borst, G. Stansby, J.D. Beard, S.T. Engelter, T. Richards, M.M. Brown, ICSS Investigators

**Affiliations:** a Institute of Neurology, University College London, UK; b Department of Biostatistics and Bioinformatics and Duke Global Health Institute, Duke University, Durham, NC, USA; c Department of Medical Statistics, London School of Hygiene and Tropical Medicine, UK; d Department of Vascular Surgery, University Medical Centre Utrecht, The Netherlands; e Department of Vascular Surgery, Freeman Hospital, Newcastle upon Tyne, UK; f Sheffield Vascular Institute, Northern General Hospital, Sheffield, UK; g Department of Neurology and Stroke Center, University Hospital Basel, Basel, Switzerland; h Division of Surgery and Interventional Science, University College London, UK

**Keywords:** Carotid atherosclerosis, Carotid artery stenosis, Carotid endarterectomy

## Abstract

**Objectives:**

Carotid endarterectomy (CEA) is standard treatment for symptomatic carotid artery stenosis but carries a risk of stroke, myocardial infarction (MI), or death. This study investigated risk factors for these procedural complications occurring within 30 days of endarterectomy in the International Carotid Stenting Study (ICSS).

**Methods:**

Patients with recently symptomatic carotid stenosis >50% were randomly allocated to endarterectomy or stenting. Analysis is reported of patients in ICSS assigned to endarterectomy and limited to those in whom CEA was initiated. The occurrence of stroke, MI, or death within 30 days of the procedure was reported by investigators and adjudicated. Demographic and technical risk factors for these complications were analysed sequentially in a binomial regression analysis and subsequently in a multivariable model.

**Results:**

Eight-hundred and twenty-one patients were included in the analysis. The risk of stroke, MI, or death within 30 days of CEA was 4.0%. The risk was higher in female patients (risk ratio [RR] 1.98, 95% CI 1.02–3.87, *p* = .05) and with increasing baseline diastolic blood pressure (dBP) (RR 1.30 per +10 mmHg, 95% CI 1.02–1.66, *p* = .04). Mean baseline dBP, obtained at the time of randomization in the trial, was 78 mmHg (SD 13 mmHg). In a multivariable model, only dBP remained a significant predictor. The risk was not related to the type of surgical reconstruction, anaesthetic technique, or perioperative medication regimen. Patients undergoing CEA stayed a median of 4 days before discharge, and 21.2% of events occurred on or after the day of discharge.

**Conclusions:**

Increasing diastolic blood pressure was the only independent risk factor for stroke, MI, or death following CEA. Cautious attention to blood pressure control following symptoms attributable to carotid stenosis could reduce the risks associated with subsequent CEA.

**What this paper adds**

The International Carotid Stenting Study (ICSS) compared carotid artery stenting with CEA for patients with recently symptomatic carotid stenosis. The aim of the present study was to determine whether there were subgroups of surgical patients in ICSS at higher risk of stroke, myocardial infarction, or death, and whether specific surgical factors are associated with higher risk. It was found that increasing diastolic blood pressure was the only independent risk factor. Cautious attention to blood pressure control following symptoms attributable to carotid stenosis could reduce the risks associated with subsequent CEA.

## Introduction

Three major trials of carotid surgery versus best medical therapy for symptomatic carotid stenosis (the North American Symptomatic Carotid Study, NASCET,^1^ the European Carotid Surgery Trial, ECST,^2^ and the smaller Veteran's Affairs Trial)^3^ demonstrated the benefit of carotid endarterectomy (CEA) in reducing the long-term rate of recurrent stroke.^4^ Since these trials published their results, CEA has become the standard of care for patients with >50% symptomatic carotid stenosis. However, despite developments in secondary prevention medical therapy, anaesthetic technique, surgical technique, and processes of care, there remains a significant risk of major complications associated with CEA.^5^ Trials have focussed on the endpoints of stroke, myocardial infarction (MI), and death. Stroke and MI have a significant adverse impact on the patient's long-term survival – in-hospital stroke in particular has been shown in one study to confer a two-fold lower survival in the first year after surgery.^6^

There is variability in surgical technique for CEA^7^, ^8^ and debate remains over optimal processes of care, including perioperative antiplatelet therapy, type of arterial reconstruction (standard, patch, or eversion CEA) and mode of anaesthesia (general, local, or combined local-general anaesthesia).

The International Carotid Stenting Study (ICSS) was an international multicentre randomized controlled open clinical trial that compared the newer technique of carotid artery stenting (CAS) with CEA for patients with recently symptomatic carotid stenosis. This study aimed to determine whether there were subgroups of surgical patients in ICSS at higher risk of stroke, MI, or death, and whether specific surgical factors are associated with higher risk.

## Method

### Patient selection and protocol design

The trial protocol for ICSS is published elsewhere.^9^ In summary, patients aged >40 years were eligible for randomization in ICSS if they experienced symptoms within the 12 months before randomization attributable to a >50% diameter-reducing stenosis in the region of the common carotid artery bifurcation caused by atheromatous disease. They were required to be able to undergo either CAS or CEA. Patients were excluded if they would not be suitable for surgery because of a surgically inaccessible distal stenosis or hostile neck, had a major stroke with poor recovery of function, if they were clinically unstable (e.g. had progressive symptoms), if their vascular anatomy rendered CAS or CEA unsuitable, if cardiac bypass was planned within 1 month of the revascularization procedure, or if there had been previous revascularization of the symptomatic artery. The study was approved by ethics committees at local sites and the Northwest Multicentre research ethics committee in the UK.

Carotid endarterectomy in ICSS was performed according to the surgeon's usual practice: local, general, or combined anaesthesia was allowed for the procedure. The type of arterial reconstruction to be carried out was not specified in the protocol, nor was a specific peri-procedural medication regimen. The use of shunts or patches was optional. However, all patients were required to receive “best medical therapy,” including antiplatelet agents or anticoagulation where appropriate, and control of vascular risk factors. In addition to collecting the above technical information, centres supplied demographic information about the patient at the time of enrolment into the trial, and specified whether their general policy was to send patients to a specialized post-procedure ward following CEA, such as an intensive care unit, or a general surgical or medical ward.

Only patients assigned to CEA in ICSS in whom CEA was initiated were included. Initiation of CEA was defined as the administration of either local or general anaesthesia prior to commencement of surgery. Patients in whom CEA was abandoned after administration of anaesthesia were included in the analysis. Patients who crossed over without CEA or who received CEA after an attempt at stenting were excluded.

### Outcome events

Patients underwent face-to-face follow-up by a trial investigator – a neurologist or physician interested in stroke – at 30 days after surgery. Stroke, MI, or death occurring within 30 days of the procedure was reported to the central trial office by investigators. Stroke was defined as “an acute disturbance of focal neurological function lasting more than 24 hours resulting from intracranial vascular disturbance.” A diagnosis of MI required two of the following: cardiac enzymes more than twice the upper limit of normal, a history of chest discomfort lasting at least 30 minutes, or the development of specific ECG abnormalities.

Outcome events were reported in detail to the central office by the local neurologist or stroke physician, along with confirmatory evidence (e.g. CT/MRI, blood test results, or death certificate) where available. Major outcome events were submitted to an independent external adjudicator, who was masked to treatment allocation and who determined the cause, severity, and duration of the event. If this assessment differed from the initial assessment, a second external adjudicator reviewed the event and any differences were resolved by consensus.

### Role of the funding source

The trial funders had no role in the design of ICSS or this analysis, data collection, drafting of the manuscript, or the decision to publish.

### Statistical analysis

Risk factors for the combined outcome of stroke, MI, or death were examined sequentially in univariable binomial regression analysis using maximum likelihood estimation. Subsequent events within 30 days of the procedure were not included in the analysis. Patients with missing data were excluded from each relevant analysis. The risk ratio for each factor was estimated with a 95% confidence interval. Wald tests were used for continuous and binary predictors, with an overall likelihood ratio test for categorical predictors of more than two levels. A multivariable model was developed using a forward stepwise approach. Analyses were performed using Stata (Stata Statistical Software: Release 12, StataCorp 2011, College Station, TX).

### Clinical trial registration

ICSS is a registered clinical trial: ISRCTN 25337470 (http://www.controlled-trials.com/ISRCTN25337470).

## Results

### Occurrence and timing of events

Of the patients randomized in ICSS (1713), 858 were allocated to surgery. One subsequently withdrew all consent immediately after randomization, 15 crossed over to stenting, and 21 underwent no procedure. CEA was therefore initiated in 821 (96.0%) patients allocated to the surgery. Two patients then had their procedure aborted. Within 30 days of the procedure, 27/821 (3.3%) patients suffered a stroke of any severity (21 ischaemic, 5 haemorrhagic, and 1 of uncertain type). Three of these strokes were fatal. One patient who had a postoperative non-disabling stroke subsequently had a disabling stroke within 30 days, and another experienced two postoperative non-disabling strokes within 30 days. Of these strokes, 25/27 were ipsilateral to the artery being treated. No patient had a retinal stroke. Non-fatal MI occurred in 5/821 patients (0.6%), and one (0.1%) patient died from another cause. The combined risk of stroke, MI, or death within 30 days of their procedure was 4.0% (33/821). Of these events, 13/33 (39.4%) occurred on day 0 – the day of the procedure. Patients undergoing CEA stayed a median of 4 days before discharge, and 7/33 (21.2%) events occurred on or after their date of discharge.

### Patient characteristics

**Figure 1:**
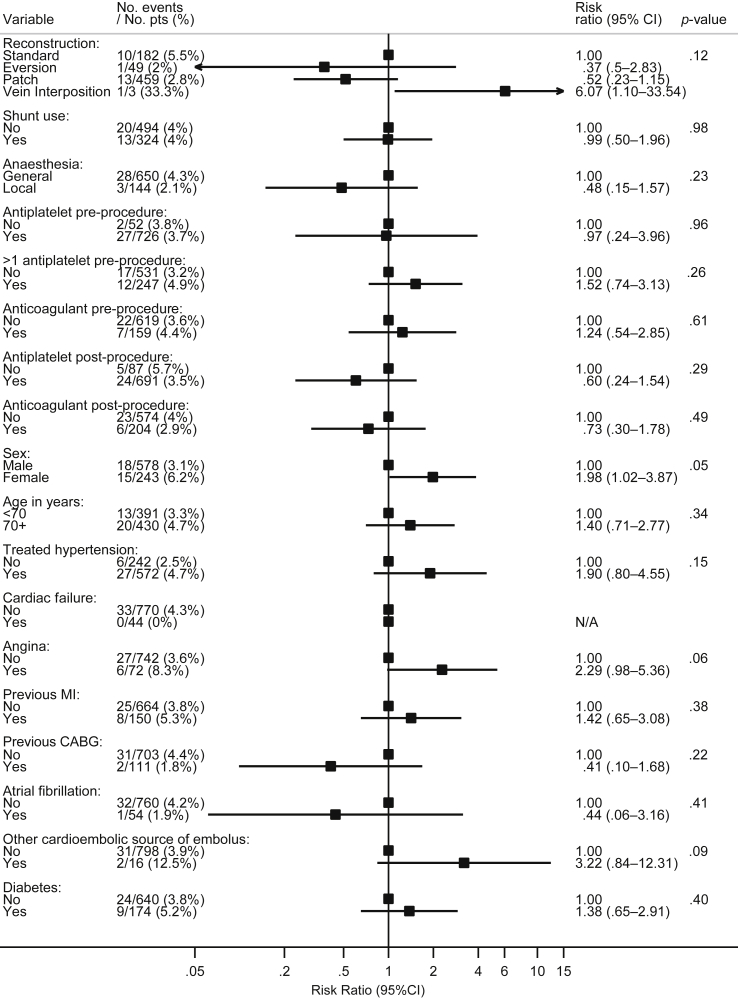

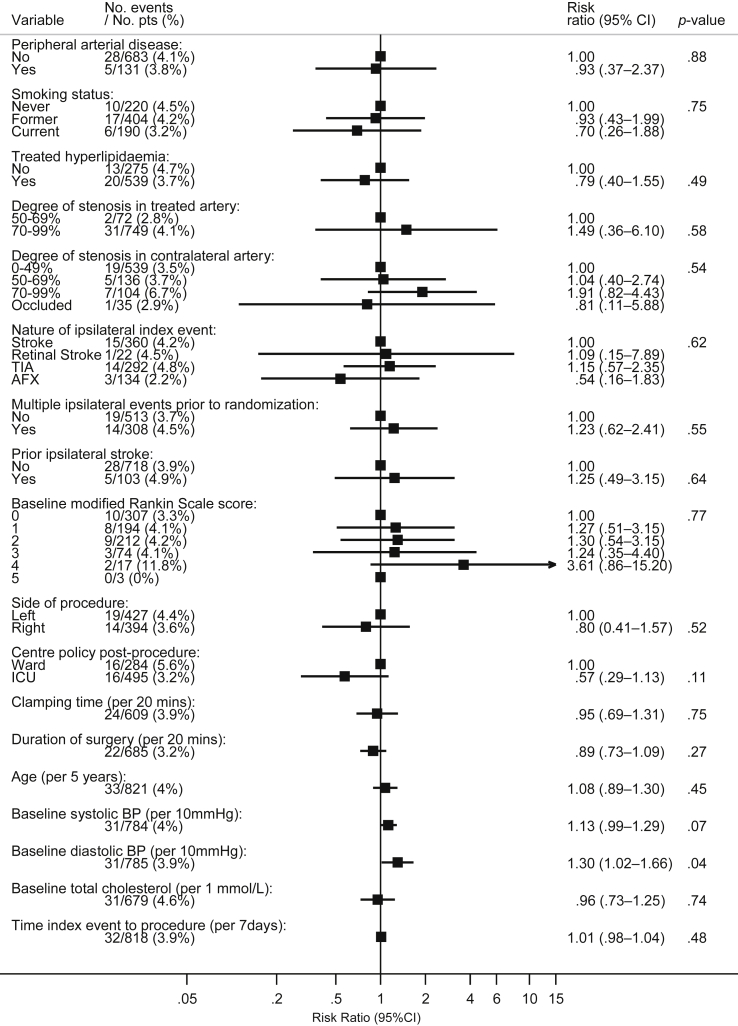
Univariable predictors of risk of stroke, death, or myocardial infarction in 821 patients undergoing CEA in whom the procedure was initiated. AFX, amaurosis fugax; BP, blood pressure; CABG, coronary artery bypass graft; ICU, intensive care unit; MI, myocardial infarction; TIA, transient ischaemic attack.

Patient and procedure characteristics are summarized in Fig. 1 along with the results of the present analysis. Of the patients, 70.4% were male, and 52.4% were aged >70 years. Vascular risk factors were common, with treated hyperlipidaemia in 65.7% and diabetes in 21.2%. Of the patients, 72.4% were either current or ex-smokers. Summary statistics for continuous variables in the analysis are given in Table 1.

### Characteristics of the procedure

General or combined local-general anaesthesia was administered to 79.2% patients undergoing CEA, and a shunt was used in 39.5%. The type of arterial reconstruction was “standard” primary closure in 22.1%, patch closure in 55.9%, and eversion endarterectomy in 6.0%, while vein interposition was used in only three patients (0.4%) (data were missing for the remaining patients). Of the patients, 726/821 (88.4%) were taking an antiplatelet agent prior to the procedure, of whom 247 (34.0%) were prescribed dual antiplatelet therapy.

### Risk factors for stroke, MI, or death

The results of univariable analyses are presented in Fig. 1. The risk of the combined outcome of stroke, MI, or death within 30 days of the procedure was significantly higher in female patients (risk ratio [RR] 1.98, 95% CI 1.02–3.87, *p* = .05). Diastolic blood pressure also significantly predicted risk (RR 1.30 for each +10 mmHg, 95% CI 1.02–1.66, *p* = .04). Mean baseline diastolic blood pressure, obtained at the time of randomization in the trial, was 78 mmHg (SD 13 mmHg).

The median time from index event, prompting randomization in the trial, to CEA was 40 days (IQR 18–87 days), and 18% underwent CEA within 14 days of the index event. The time from the index event to the date of surgery significantly predicted the risk of the combined outcome of stroke, MI, or death within 30 days of the procedure, but the statistical significance of this result was influenced by one outlying patient. Removing this patient in a sensitivity analysis resulted in a non-significant *p*-value (RR 1.01 per 7 days, 95% CI 0.98–1.04, *p* = .48).

There was no evidence that other baseline variables including age (RR 1.08 per 5 years, 95% CI 0.89–1.30, *p* = .45), the patient's level of disability (*p* = .77), or the degree of ipsilateral (*p* = .58) or contralateral carotid stenosis (*p* = .54) were statistically significant predictors of risk.

Shunt use (RR 0.99, 95% CI 0.50–1.96, *p* = .98) and local anaesthesia only versus general or combined (RR 0.48, 95% CI 0.15–1.57, *p* = .23) did not significantly predict risk. The overall comparison between types of reconstruction (standard, eversion, patch and vein interposition) was not significant (*p* = .12). There was a decrease in the risk of an event in centres with a policy of sending the patient to an intensive care or other specialized post-procedure unit for 1 hour or more (RR 0.57, 95% CI 0.29–1.13, *p* = .11), although this result was not statistically significant.

**Figure 2:**
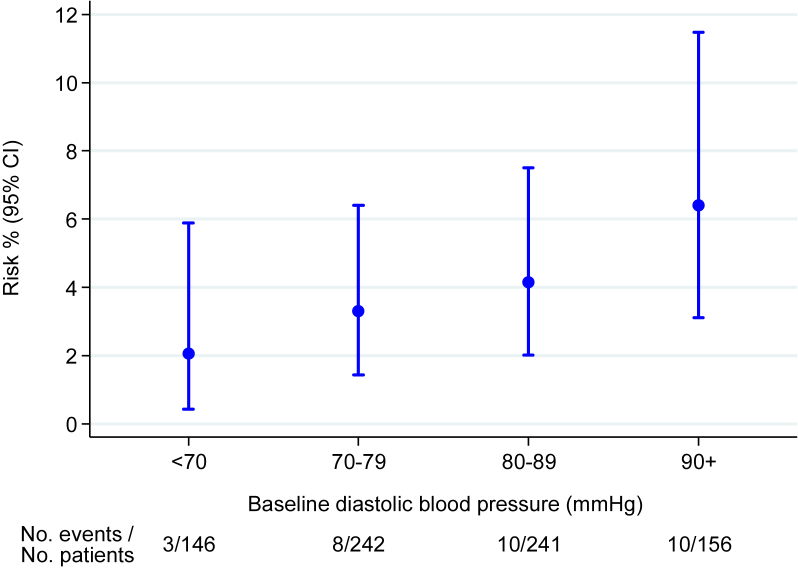
Observed risk (95% confidence interval) of stroke, MI, or death with increasing categories of baseline diastolic blood pressure.

In the multivariable analysis, only baseline diastolic blood pressure, which was available for 785/821 (95·6%) patients, remained a significant predictor of risk and thus the estimated risk ratio is unchanged from the univariable analysis (RR 1.30 for each +10 mmHg, 95% CI 1.02–1.66, *p* = .04). Fig. 2 illustrates the increase in the observed risk of the combined endpoint of stroke, MI, or death in ICSS patients for patients with increasing baseline diastolic blood pressure.

## Discussion

### Summary

Of the baseline demographic and vascular risk factor variables examined, only sex and baseline diastolic blood pressure significantly predicted the risk of the combined outcome of stroke, MI, or death within 30 days of CEA, with female patients having approximately double the risk of male patients. After adjustment in a multivariable model, only baseline diastolic blood pressure remained a significant predictor of risk. There was no evidence that the surgical technical variables, including type of anaesthesia, use of a shunt, or type of surgical reconstruction significantly influenced risk.

### Research in context

The finding that symptomatic women undergoing CEA in ICSS experienced a higher risk of stroke, MI, or death than men is broadly consistent with findings from other randomized trials and registry data of carotid endarterectomy,^4^, ^10^, ^11^, ^12^ although more recent data from the Society for Vascular Surgery Vascular Registry suggest a similar complication risk (around 4% risk of stroke, MI, or death within 30 days of CEA).^13^ One potential explanation for this may be that a smaller average carotid artery diameter in women is associated with procedural stroke caused by in-situ thrombosis following more technically demanding surgery.^14^, ^12^ However, sex was not an independent predictor of risk in the present multivariable analysis, suggesting that differences in other baseline patient characteristics between male and female patients could account for the increased operative risk in women.^15^

The association between raised baseline blood pressure and outcome of surgery has also been demonstrated previously in a systematic review that included patients from the European Carotid Surgery Trial.^16^ In ICSS, 8.3% of patients undergoing CEA experienced post-procedural hypertension which was associated with higher baseline blood pressure.^17^ Other authors have shown an association between preoperative blood pressure, postoperative blood pressure, and stroke or death.^18^, ^19^ However, it is notable that all of these previous studies of blood pressure and the risk of CEA reported only systolic blood pressure and none reported an analysis of diastolic blood pressure, perhaps because of an assumption that systolic blood pressure would be a better predictor of risk. The present study suggests that in the context of a single preoperative measurement of blood pressure, diastolic blood pressure might be the better predictor. Given the consistency of raised blood pressure as a risk factor for perioperative events, consideration should be given to cautious lowering of raised blood pressure prior to CEA. However, it should be borne in mind that overzealous lowering of blood pressure might be hazardous in patients with impaired cerebral perfusion, for example in those with border zone ischaemia or contralateral occlusion.

Time from index symptom to procedure did not appear to influence the risk of surgical complications. However, most patients in ICSS underwent CEA more than 14 days after randomization,^20^ whereas today there is an increasing trend to operate on patients much earlier. In a 2012 UK audit, one-third of patients underwent CEA within 14 days of symptoms,^7^ but there is some evidence from the large Swedish Vascular Registry that the death and stroke rate among patients operated on within 48 hours of a qualifying event is increased.^21^ The risk factors for stroke occurring in patients operated within a few days of symptoms might be different to those found in the present study.

### Limitations

The risk of stroke, MI, or death within 30 days of surgery in the symptomatic CEA patients was acceptably low; however, the low number of events limited ability to detect more moderate risk factors. This also limited the number of variables supported in a multivariable model.

Despite detailed individual patient measurements in ICSS, only baseline and discharge blood pressures were collected and therefore it was not possible to assess the influence of perioperative haemodynamic control on outcome. Moreover, the protocol did not define the method or timing of baseline blood pressure measurements, which might have resulted in more blood pressure variability than a standardized technique. It is also possible that more generalized measures of physiological health, such as the patient's American Society of Anesthesiology (ASA) grade would be a better predictor of adverse outcomes following CEA.^22^ Only 18% of patients in the present study group received CEA within 2 weeks of symptoms, and therefore results might not apply to patients treated soon after symptoms.

### Conclusion

Patients with higher baseline diastolic blood pressures undergoing CEA in ICSS were at higher risk of stroke, MI, or death within 30 days of the procedure. ICSS showed surgery was safer compared with previous randomized trials of symptomatic patients undergoing CEA, which might have limited ability to detect other predictors of risk. Nonetheless, cautious attention to blood pressure control after stroke or TIA might help to reduce the risk of serious complications in patients undergoing subsequent CEA. The finding that approximately one-fifth of patients with an event experienced this on or after the date of discharge suggests a need for careful post-surgical follow-up and attention to other vascular risk factors.

## Conflict of Interest

None.

## Funding

ICSS was funded by the UK Medical Research Council (MRC, grant number G0300411) and managed by the UK National Institute for Health Research (NIHR) on behalf of the MRC-NIHR partnership. The views and opinions expressed herein are those of the authors and do not necessarily reflect those of the NIHR Health Services Research programme of the Department of Health. Additional funding was supplied by grants from the Stroke Association (grant number TSA 2005/01 and TSA 2007/12), Sanofi-Synthelabo and the European Union. MMB's Chair in Stroke Medicine is supported by the Reta Lila Weston Trust for Medical Research. DD and RLF were supported by the grant from the MRC. This work was undertaken at University College London, which received a proportion of funding from the UK Department of Health's National Institute for Health Research Biomedical Research Centres funding scheme.

## Figures and Tables

**Table 1:** Summary statistics for baseline age, systolic blood pressure, diastolic blood pressure, total cholesterol, time from event to index procedure, duration of surgery, and clamping time in 821 patients undergoing CEA.

Patient characteristic	No. events/No. patients^a^	Mean (SD) or median (IQR)
Baseline age in years	33/821	70 (9)
Baseline systolic blood pressure in mmHg	31/784	146 (24)
Baseline diastolic blood pressure in mmHg	31/785	78 (13)
Baseline total cholesterol in mmol/L	31/679	4.9 (1.3)
Time index event to procedure in days	32/818	40 (18–87)
Duration of surgery in minutes	22/685	103 (79–130)
Clamping time in minutes	24/609	23 (7.5–35)

a Patients with available data.
